# Co-Morbidity between Early-Onset Leukemia and Type 1 Diabetes – Suggestive of a Shared Viral Etiology?

**DOI:** 10.1371/journal.pone.0039523

**Published:** 2012-06-22

**Authors:** Kari Hemminki, Richard Houlston, Jan Sundquist, Kristina Sundquist, Xiaochen Shu

**Affiliations:** 1 Division of Molecular Genetic Epidemiology, German Cancer Research Center, Heidelberg, Germany; 2 Center for Primary Health Care Research, Lund University, Malmö, Sweden; 3 Section of Cancer Genetics, Institute of Cancer Research, Sutton, United Kingdom; 4 Stanford Prevention Research Center, Stanford University School of Medicine, Stanford, California, United States of America; The Catalan Institute of Oncology (ICO), Spain

## Abstract

**Background:**

Acute lymphoblastic leukemia (ALL) and acute myeloid leukemia (AML) are common early-onset malignancies. Their causes are largely unknown but infectious etiology has been implicated. Type 1 diabetes (T1D) is an autoimmune disease for which infectious triggers of disease onset have been sought and increasing pointing to enteroviruses. Based on our previous results on co-morbidity between leukemia and T1D, we updated the Swedish dataset and focused on early onset leukemias in patients who had been hospitalized for T1D, comparing to those not hospitalized for T1D.

**Methods and Findings:**

Standardized incidence ratios (SIRs) were calculated for leukemia in 24,052 patients hospitalized for T1D covering years 1964 through 2008. T1D patients were included if hospitalized before age 21 years. Practically all Swedish children and adolescents with T1D are hospitalized at the start of insulin treatment. SIR for ALL was 8.30 (N = 18, 95% confidence interval 4.91–13.14) when diagnosed at age 10 to 20 years after hospitalization for T1D and it was 3.51 (13, 1.86–6.02) before hospitalization for T1D. The SIR for ALL was 19.85 (N = 33, 13.74–27.76) and that for AML was 25.28 (8, 10.80–50.06) when the leukemias were diagnosed within the year of T1D hospitalization. The SIRs increased to 38.97 (26, 25.43–57.18) and 40.11 (8, 17.13–79.42) when T1D was diagnosed between ages 10 to 20 years. No consistent time-dependent changes were found in leukemia risk.

**Conclusion:**

A shared infectious etiology could be a plausible explanation to the observed co-morbidity. Other possible contributing factors could be insulin therapy or T1D related metabolic disturbances.

## Introduction

Acute lymphoblastic leukemia (ALL) is the most common childhood malignancy. The underlying causes are largely unknown beyond ionizing radiation and the recently detected susceptibility genes [1], [2], [3]. However, an infectious etiology has been strongly implicated but the agents have evaded identification [4], [5], [6], [7], [8]. Acute myeloid leukemia (AML) is an adult disease but a few percent of cases occur in the childhood [9]. Viruses, solvents, ionizing radiation and smoking have been implicated in adults but the causes of childhood cases are unknown [9]. Type 1 diabetes mellitus (T1D) is an early-onset autoimmune disease in which immune response is directed against the insulin-producing beta cells of the pancreas, resulting in insulin-dependence. The autoimmune process is thought to be triggered in susceptible individuals by unknown factors [10], [11]. T1D is increasingly thought to have a viral basis, probably a consequence through genetically determined aberrant response to an enteroviral infection [12], [13]. Enterovirus RNA has been demonstrated in the blood and pancreatic tissue of early onset T1D patients [12]. Family history is an important risk factor for T1D for which the HLA region is the most important contributor [13], [14], [15]. The HLA locus regulates the response to many viral infections and even other T1D susceptibility loci are relevant to antiviral actions, including the IFIH1 gene modulating innate immunity [12]. Recently, genetic variation around the gene encoding IKZF1, a regulator of lymphocyte development and immune homeostasis, has been shown to confer susceptibility to both childhood ALL and T1D [1], [16]. Previous epidemiological studies have shown significant correlations in incidence between ALL and T1D at both the international and regional level, with potential but unproven links with an infectious etiology [17], [18].

In a previous study of cancer risk in T1D patients, we reported increased risk of leukemia, stomach and skin cancers [19]. In view of the increasing data on the possible viral etiology of T1D, we updated the Swedish dataset and investigated in detail the associations of early-onset leukemia with T1D.

## Methods

The analyses were carried out by linking T1D patients (N = 24,052) from the nation-wide Hospital Discharge Registry (years 1964 to 2008) with cancer data from the Swedish Cancer Registry, collected in the Family-Cancer Database (1964 to 2008) [19]. It should be noted that in the previous study the population of T1D patients was as in the present study but cancer follow-up was extended by 2 more years for the present study [19]. Practically all Swedish children and adolescents with T1D are hospitalized at the start of insulin treatment [20], hence selection bias is not a source of confounding. Also the diagnostic accuracy is high [21]. T1D was defined to include patients whose age at first hospitalization for diabetes was before age 21. In the previous study we assessed how well this age limit was able to discriminate T1D from other types of diabetes. According to International Classification of Diseases version 10, listing T1D as an independent disease, 98% of diabetics diagnosed before age 21 years were T1D patients [19].

Person-years were calculated from the start of follow-up at hospitalization for T1D until diagnosis of cancer, death, emigration, or closing date on December 31, 2008. Alternative analyses considered leukemia diagnosed before hospitalization for T1D or that T1D and leukemia were diagnosed within one year of each other. Standardized incidence ratios (SIRs) were calculated as the ratio of observed (O) to expected number of cases. Expected rates were calculated for age (10-year groups), gender, period of diagnosis (10-year bands) and region (4 groups) and compared with individuals without hospitalization for T1D [19]. As T1D is a rare disease, the expected rate without hospitalization for T1D was essentially the population rate.

The study was approved by the ethics committee at Lund University.

## Results

SIRs for ALL, AML, chronic lymphocytic leukemia (CLL) and chronic myeloid leukemia (CML) following hospitalization for T1D are detailed in Table 1. SIRs were significantly increased for both ALL (5.70, 3.68–8.43) and AML (2.57, 1.02–5.32) but not for either CLL or CML. The highest SIR for ALL of 8.30 (4.91–13.14) was attained for a diagnosis between age 10 and 20 years following hospitalization for T1D. When diagnosed before hospitalization for T1D, the SIR for ALL was 3.51 (1.86–6.02) and that for AML was 4.80 (1.52–11.30) at age 10 to 20 years. Overall across all ages the SIRs for ALL and AML were highly significant at age 10 to 20 years (5.37, 3.65–7.64 and 4.50, 1.92–8.92) and at age 0 to 50 years (4.17, 3.07–5.53 and 3.77, 2.05–6.34). While stratification by sex showed higher ALL rates for females and higher AML rates for males, all the key findings were significant for both males and females (data not shown).

**Table 1:** SIRs for leukemia in T1DM patients according to age at leukemia diagnosis.

Leukemia	Age at diagnostic for leukemia (years)															
	<10				10 – 20				21 – 50				All			
	O	SIR	95%	CI	O	SIR	95%	CI	O	SIR	95%	CI	O	SIR	95%	CI
**Leukemia diagnosed after hospitalization for T1D**																
ALL	3	2.31	0.43	6.83	18	8.30**	4.91	13.14	4	3.63	0.95	9.40	25	5.70**	3.68	8.43
CLL	0				0				3	9.48*	1.79	28.05	3	4.09	0.77	12.11
AML	0				3	3.97	0.76	11.75	4	2.31	0.60	5.97	7	2.57*	1.02	5.32
CML	0				0				3	2.33	0.44	6.89	3	1.70	0.32	5.03
**Leukemia diagnosed before hospitalization for T1D**																
ALL	10	1.60	0.76	2.94	13	3.51**	1.86	6.02	0				23	3.22**	2.04	4.84
CLL	0				0				0				0			
AML	2	2.33	0.22	8.58	5	4.80*	1.52	11.30	0				7	6.91**	2.74	14.31
CML	0				0				0				0			
**Leukemia diagnosed before or after hospitalization for T1D**																
ALL	13	1.72	0.91	2.95	31	5.37**	3.65	7.64	4	3.63	0.95	9.40	48	4.17**	3.07	5.53
CLL	0				0				3	9.48*	1.79	28.05	3	4.09	0.77	12.11
AML	2	2.33	0.22	8.58	8	4.50*	1.92	8.92	4	2.31	0.60	5.97	14	3.77*	2.05	6.34
CML	0				0				3	2.33	0.44	6.89	3	1.70	0.32	5.03

O, observed; SIR, standardized incidence ratio; CI, confidence interval; *P<0.05, **P<0.01.

Case numbers in the reference population ALL: 1733 (<10 y), 701 (10–20 y), 562 (21–50 y), 2996 (total); CLL: 55 (<10 y), 15 (10–20 y), 728 (21–50 y), 798 (total); AML: 301 (<10 y), 296 (10–20 y), 1506 (21–50 y), 2103 (total); CML: 88 (<10 y), 93 (10–20 y), 1201 (21–50 y), 1382 (total).

In Table 1, the highest risks of ALL and AML were observed in patients first hospitalized for T1D at age 10 to 20 years which age group also showed the highest incidence for T1D hospitalization. Considering mean ages for T1D hospitalization for ‘leukemia diagnosis before or after hospitalization for T1D’ in Table 1, for ALL these were 7.6 years (ALL diagnosis <10 years), 14.3 years (ALL diagnosis 10–20 years) and 12.8 years (ALL diagnosis 21–50 years); the corresponding median ages for T1D hospitalization relating to AML were 11.5, 16.9 and 15.3 years.

**Figure 1:**
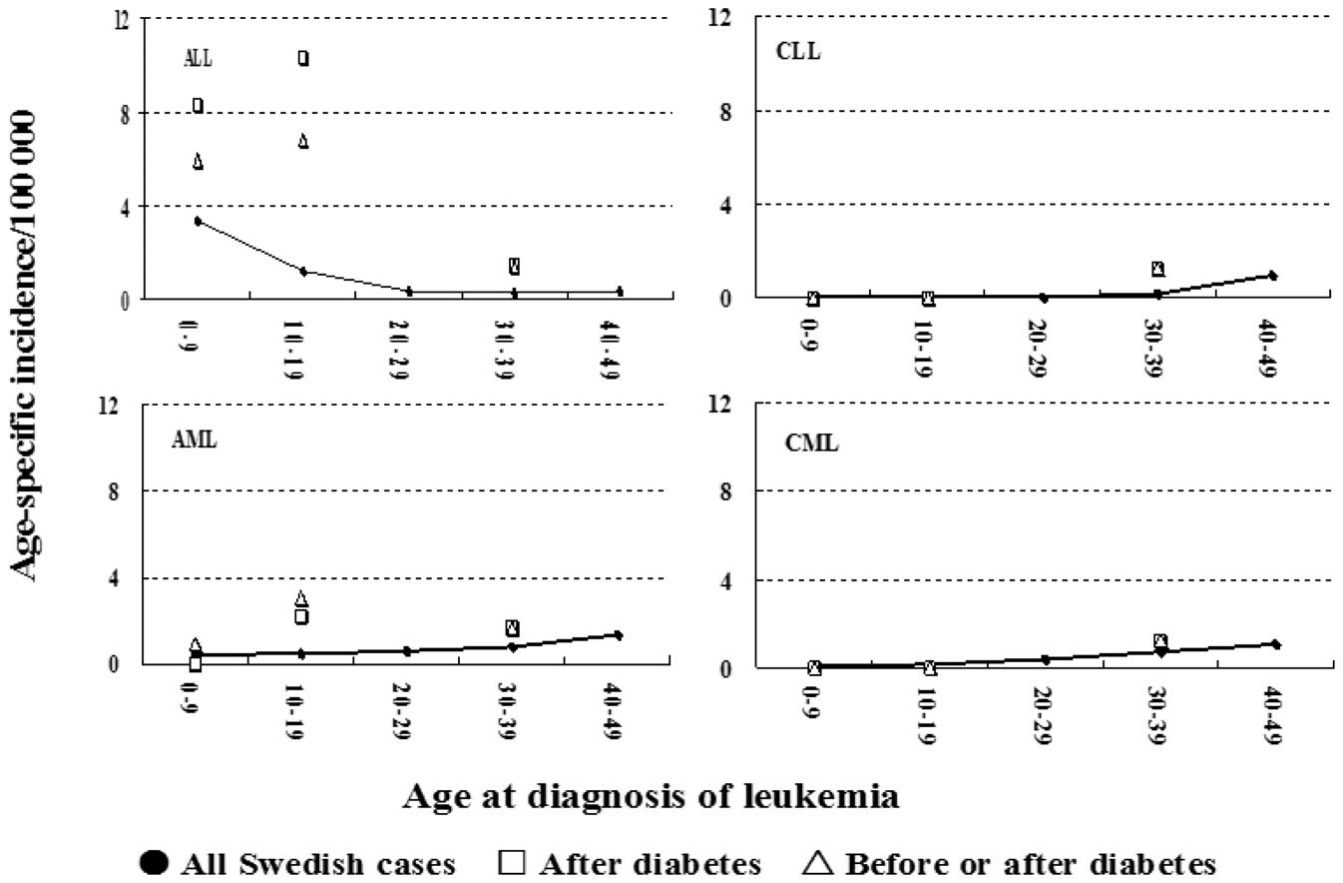
Age-specific incidence of leukemia in T1D patients (symbols in three age bands, 0–9 y, 10–19 y and 20–49 y) compared to the Swedish background rate (solid lines in 10–year age bands). The case numbers for T1D patients are shown in Table 1 and those for the background rates in footnote to Table 1.

Age-specific incidence of leukemia in T1D patients is shown in Fig. 1 in comparison to the Swedish background rates. The incidence in ALL among T1D patients was essentially equal in age bands 0–9 y and 10–19 y but because the background incidence declined with age the SIRs were higher for the latter age group as was shown in Table 1. For AML, the highest incidence in T1D patients was in age group 10–19 y.

The SIRs were very high if leukemia was diagnosed within one year of hospitalization for T1D; the SIR for ALL was 28.59 (15.95–47.27) and that for AML was 28.50 (5.37–84.36)(Table 2). They were even higher, 72.87 (39.70–122.59) and 43.27 (8.16–128.09), when T1D patients were hospitalized at age 10 to 20 years. In contrast, if events were separated by more than one year, the respective SIRs were 2.59 (1.23–4.77) and 1.55 (0.40–4.02). When hospitalized for T1D before age 10, the only significant increase of 5.85 was for ALL diagnosed within one year (N = 7, 95%CI 2.49–11.27, before or after T1D hospitalization) (data not shown).

**Table 2:** SIRs for leukemia in T1D patients according to time at hospitalization for T1D and age at T1D diagnosis.

Leukemia(T1D diagnosis at age10–20 years)		Time at hospitalization for T1D (years)							
		Within 1 year				More than 1 year			
		O	SIR	95%	CI	O	SIR	95%	CI
		**Leukemia diagnosed after hospitalization for T1D**							
ALL		15	28.59**	15.95	47.27	10	2.59*	1.23	4.77
ALL	(T1D 10-20 y)	14	72.87**	39.70	122.59	6	3.58*	1.29	7.85
AML		3	28.50**	5.37	84.36	4	1.55	0.40	4.02
AML	(T1D 10-20 y)	3	43.27**	8.16	128.09	4	2.11	0.55	5.45
		**Leukemia diagnosed before hospitalization for T1D**							
ALL		18	15.99**	9.61	25.02	5	0.76	0.24	1.79
ALL	(T1D 10-20 y)	12	25.26**	12.99	44.27	3	0.62	0.12	1.84
AML		5	23.68	7.47	55.69	2	2.20	0.21	8.07
AML	(T1D 10-20 y)	5	38.42	12.12	90.38	1	1.43	0.00	8.21
		**Leukemia diagnosed before OR after hospitalization for T1D**							
ALL		33	19.85**	13.74	27.76	15	1.38	0.95	2.97
ALL	(T1D 10-20 y)	26	38.97**	25.43	57.18	9	1.58	0.89	4.77
AML		8	25.28**	10.80	50.06	6	1.77	0.56	3.41
AML	(T1D 10-20 y)	8	40.11**	17.13	79.42	5	1.87	0.49	5.89

O, observed; SIR, standardized incidence ratio; CI, confidence interval;

* P<0.05, **P<0.01.

According to Table 2, the majority of T1D hospitalizations and leukemia diagnoses coincided with the same year. The mean calendar time difference between the two events depended however on the age at T1D hospitalization. For ‘leukemia diagnosed before or after hospitalization for T1D’ the median time difference was only 0.8 years for ALL and 0.7 year for AML in the most common leukemia diagnostic group of 10 to 20 years. For leukemias diagnosed <10 year the mean difference was 3.5 years for ALL and 9.0 years for AML; for leukemias diagnosed between 21 and 50 years the mean difference was 13.8 years for ALL and 18.5 years for AML.

Several additional analyses were conducted (data not shown). Analyses by calendar period showed that there was no significant time trend for ALL or AML. The number of hospitalizations for T1D (1 to 3 compared to 3+) did not influence the risk for ALL (4.10, 2.72–5.93 vs 4.29, 2.62–6.64) and for AML the only significant SIR was for those hospitalized 1 to 3 times.

## Discussion

Outside therapeutic settings and exclusive of Down syndrome and co-twin affected with ALL, the present risks for ALL and AML are remarkably high [22], [23]. For example, the analysis of sibship size as a proxy of infectious etiology showed an effect for ALL but not for AML, yet the overall risk was only 1.3 (4 or more siblings vs. 1) but it increased 2.1-fold in those diagnosed below age 6 years [6]. Clearly, the present high risks require explanation, particularly as the hospitalized T1D population is not a small subgroup of all patients but practically all Swedish children and adolescents diagnosed with T1D, hospitalized at the start of insulin treatment [20]. There was also an indication that CLL, with a very different age distribution compared to T1D, might be related to T1D but with 3 cases the evidence awaits further confirmation. The main limitation of the present study is that it is purely epidemiological, lacking any data on serological analysis, but, at the same token, the diagnostic data on leukemia and T1D should be highly reliable [21], [24]. Demanding virological proof for the present study appears overwhelming because conclusive proof has yet been obtained neither for ALL nor T1D, in spite of numerous attempts [4], [7], [11].

Our findings provide strong evidence for a relationship between early-onset leukemia risk and T1D. While it is possible that insulin therapy causes leukaemigenesis directly through its growth-promotion effects, risks were also increased prior to first hospitalization for T1D. It is possible, although unlikely, that the high glucose levels and metabolic disturbance before the start of insulin therapy might trigger leukemia, in an analogous fashion to the increased risk of cancer associated with type 2 diabetes [25]. As the length of time to achieve stable glucose control (number of hospitalizations is a proxy for labile glucose levels) was not correlated with risk, this strongly argues against such an explanation. Furthermore, there was no evidence of a time trends, which should exclude strong environmental or treatment-related changes, assuming that the control of glucose levels has improved with time. Shared symptoms of generalized weakness and fatigue for childhood leukemia and T1D may contribute to the coincident detection, but there is no reason to doubt diagnostic accuracy. Whether autoimmunity itself would trigger leukemia is also a theoretical but not a likely option because it would not explain the coincident diagnosis of the two diseases and the decreasing leukemia risk past age 20 years. Finally by exclusion, our findings coupled with epidemiological and other data provide support for a common infective basis for the observed co-morbidity, which most probably could be viral in origin. It is not clear at what ages pancreatic tissue would be most vulnerable to viral damage but high positivity for enteroviral capsid proteins have been found in pancreatic islets in 72 patients with a mean age of 13 years, well within the present age range [26]. As genetic susceptibility to T1D and ALL is now well established an aberrant response to viral challenge leading to both diseases would provide an attractive basis for the observed co-morbidity.
